# Transport of Particles in Intestinal Mucus under Simulated Infant and Adult Physiological Conditions: Impact of Mucus Structure and Extracellular DNA

**DOI:** 10.1371/journal.pone.0095274

**Published:** 2014-04-22

**Authors:** Adam Macierzanka, Alan R. Mackie, Balazs H. Bajka, Neil M. Rigby, Françoise Nau, Didier Dupont

**Affiliations:** 1 Institute of Food Research, Norwich Research Park, Norwich, United Kingdom; 2 Agrocampus Ouest, UMR 1253, Rennes, France; 3 Institut National de la Recherche Agronomique, STLO, UMR 1253, Rennes, France; The University of Wollongong, Australia

## Abstract

The final boundary between digested food and the cells that take up nutrients in the small intestine is a protective layer of mucus. In this work, the microstructural organization and permeability of the intestinal mucus have been determined under conditions simulating those of infant and adult human small intestines. As a model, we used the mucus from the proximal (jejunal) small intestines of piglets and adult pigs. Confocal microscopy of both unfixed and fixed mucosal tissue showed mucus lining the entire jejunal epithelium. The mucus contained DNA from shed epithelial cells at different stages of degradation, with higher amounts of DNA found in the adult pig. The pig mucus comprised a coherent network of mucin and DNA with higher viscosity than the more heterogeneous piglet mucus, which resulted in increased permeability of the latter to 500-nm and 1-µm latex beads. Multiple-particle tracking experiments revealed that diffusion of the probe particles was considerably enhanced after treating mucus with DNase. The fraction of diffusive 500-nm probe particles increased in the pig mucus from 0.6% to 64% and in the piglet mucus from ca. 30% to 77% after the treatment. This suggests that extracellular DNA can significantly contribute to the microrheology and barrier properties of the intestinal mucus layer. To our knowledge, this is the first time that the structure and permeability of the small intestinal mucus have been compared between different age groups and the contribution of extracellular DNA highlighted. The results help to define rules governing colloidal transport in the developing small intestine. These are required for engineering orally administered pharmaceutical preparations with improved delivery, as well as for fabricating novel foods with enhanced nutritional quality or for controlled calorie uptake.

## Introduction

The last boundary between ingested food and the gastro-intestinal (GI) tract mucosa is the mucus layer. This highly complex viscoelastic medium has evolved to provide a robust barrier that can trap and immobilize potentially hazardous particulates such as bacteria but still allow the passage of nutrients to the epithelial surfaces [1]–[4]. These conflicting properties are particularly important in the small intestine where the mucus layer is thinnest [5] and the majority of nutrient absorption takes place. However, the rules governing this selective barrier function, particularly in relation to transport of particulates, remain unknown. Recent studies suggest that surface properties of model nanoparticles can impact on colloidal transport in intestinal mucus [6]. In our previous work [7], [8], we showed that adsorption of bile salts to the surface of model microparticles and partially digested emulsion droplets significantly enhanced their diffusion in the small intestinal mucus.

The small intestine is covered by a continuous layer of epithelial cells comprising mainly absorptive cells (enterocytes) and, to a lesser extent, goblet cells responsible for the assembly and active secretion of mucin glycoproteins. The main secreted mucin in the intestine is MUC2, and it is considered to be a major gel-forming component of the mucus responsible for its viscoelastic properties [9]. Moreover, the epithelium of fully-developed mammalian intestine is renewed typically every 3–5 days [10]. This continuous self-renewal is essential to maintain tissue homeostasis and eliminate damaged cells. It is achieved through a process in which epithelial cells generated from stem cells at the crypt base migrate to the tips of the villi where they are shed [11], [12]. The mechanism of cell shedding does not compromise mucosal barrier function in healthily tissue despite temporary discontinuities in the epithelial layer [11]. It has been suggested that apoptosis is a key process regulating cell number in the intestinal epithelium [13], and more recently, *in vivo* two-photon microscopy studies in mice revealed that in the majority of villus tip cells apoptosis was a consequence and not a cause of cell shedding [14]. The cells were shed at a rate greater than the migration suggesting that they were actively extruded from the monolayer. The fast turnover of the cells implies that substantial quantities of DNA can be accumulated in the mucus layer lining the epithelium of the small intestine and contribute, together with mucins, to the overall viscosity and permeability of the mucus layer.

In the intestinal epithelium, the cells are hierarchically arranged so that they become more differentiated along the crypt/villus axis [15]. However, the differentiation, development and movement of the epithelial cells towards the tips of villi advance at different rates in adult and neonate intestines, and in the latter it can result in longer times required for the self-renewal of the epithelium to be completed [16], [17]. This may also lead to a delay in the full development of the secretory function of goblet cells. These factors may lead to a different, age-specific composition and structural organization of the mucus layer in the small intestine.

The objective of this study was to determine whether there are age-related differences in structural organization and microrheological properties of small intestinal mucus. Such differences might influence the transport of particulates from the intestinal lumen through the mucus barrier to the epithelial layer of the small intestine of the human gut. As model systems, we used *ex vivo* samples of small intestinal mucus from piglets and mature pigs in order to simulate the mucus structures that can be found under infant and adult physiological conditions of the human small intestine. Mucus permeability was measured by tracking the motion of fluorescently tagged model particles using confocal microscopy. Additionally, the impact of extracellular DNA in the mucus on colloidal transport in this secretion was investigated. Our hypothesis is that differences in the structural organization of small intestinal mucus in infant and adult affect transport characteristics of post-digestion food particulates and/or colloidal delivery systems through that protective layer to the epithelial surface, and thus subsequent absorption and bioavailability of delivered nutrients and/or bioactive substances.

## Results

### Mucus Organization in the Proximal Small Intestine

**Figure 1:**
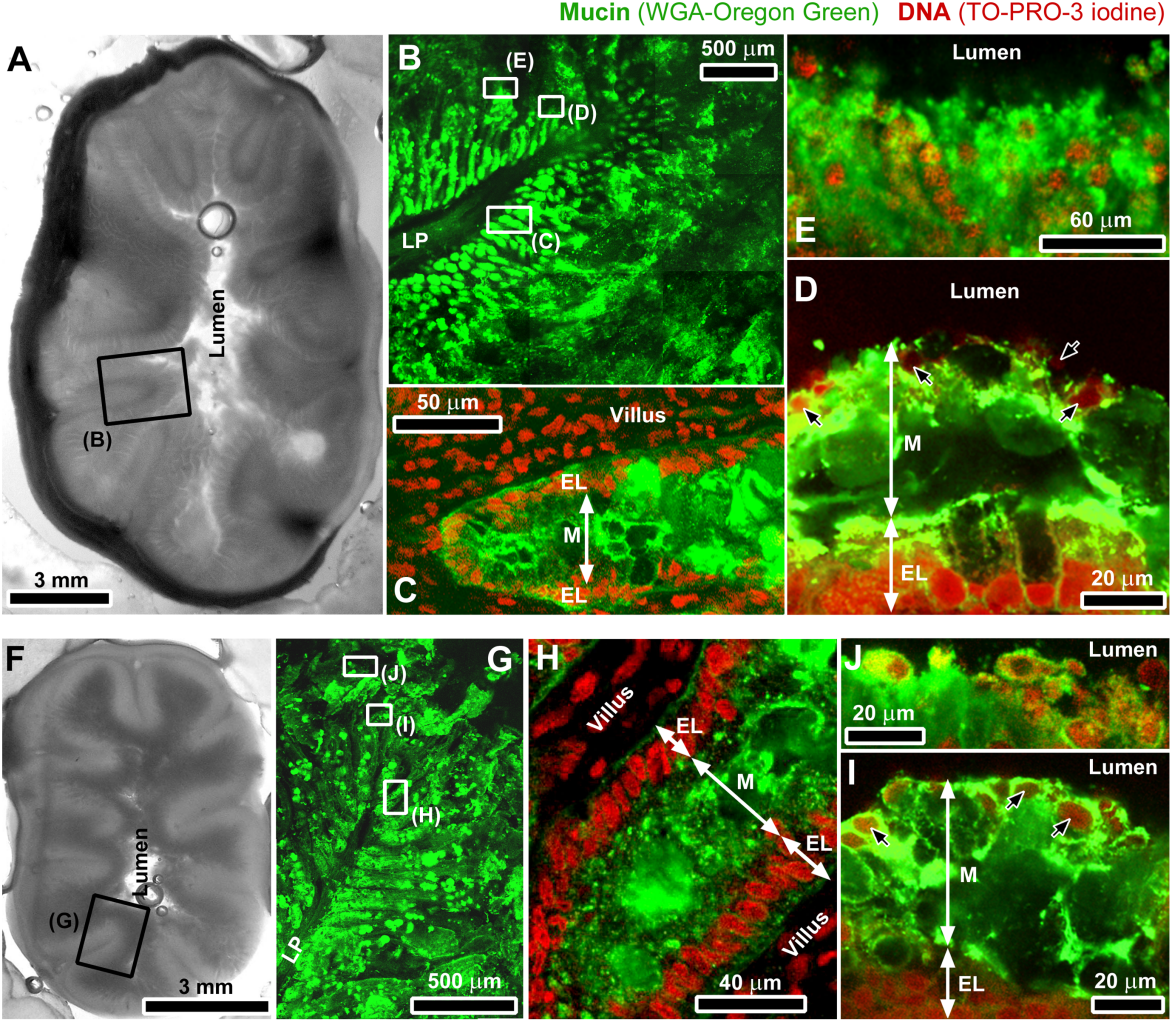
Organization of the mucus in the small intestine. Macro-scale photographs (grey-scale images) and confocal micrographs (color images) of unfixed proximal (jejunal) small intestine of pig (A–E) and piglet (F–J). The grey-scale images show complete cross-sections of the jejunal small intestine from pig (A) and piglet (F). Specimens were stained for mucin with WGA-Oregon green (green channel) and with TO-PRO-3 iodine for DNA (red channel). In the images of intestinal folds (*plicae circulares*) protruding into the lumen (B, G), the green channel for mucin was only shown. The frames highlight areas representative of those magnified in the following images. Images (C) and (H) show DNA-free mucus between villi and close to the epithelial crypts, whereas the mucus layer covering the tips of villi (D, I) contained DNA mainly from shed epithelial cells (indicated by black arrows). Images (E) and (J) show aggregates of that mucus (with both, mucin and DNA) exposed to the lumen. The symbols correspond to: LP, lamina propria, M, mucus, EL, epithelium layer.

**Figure 2:**
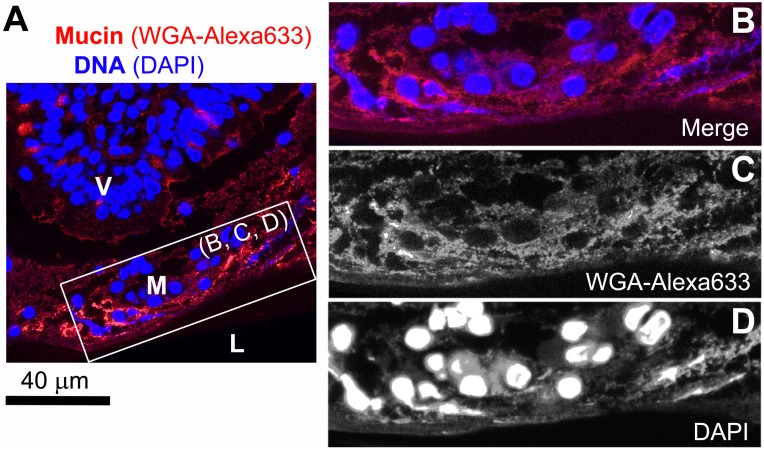
Thin section of pig small intestinal (jejunal) mucosa fixed with Carnoy’s solution. (A, B) Confocal microscopy of a villus tip (V) separated from intestinal lumen (L) with a mucus layer (M). The specimen was stained for mucin with WGA-Alexa633 and with DAPI for DNA (individual channels are shown in (B) and (C), respectively).

Prior to investigating the transport of particles in the intestinal mucus, confocal microscopy was used to visualize the architecture of the mucus in the jejunal part of pig and piglet small intestines that had been fresh-frozen after collection. Representative images have been combined in Figure 1, and show staining for mucins and DNA in sections of unfixed tissue. Despite an obvious difference in the relative diameter of the small intestine between pig and piglet (Figure 1A, F), in both cases mucus was found to cover the entire epithelial surface of the intestine and thus protect it from direct exposure to luminal contents. The mucus layer covering the tips of villi (Figure 1D, I) and the mucus released to the lumen in the form of aggregates (Figure 1E, J) contained both mucin and DNA, the latter most likely originating from degrading nuclei of the epithelial cells shed from the tips of villi as a result of the continuous turnover of the intestinal epithelium [10]. Some DNA might have also originated from intestinal bacteria. The degraded nuclei embedded in those regions of the mucus were often fragmented and deformed into irregular shapes, and ranged from the size of nuclei of the intestinal epithelial cells down to submicron particulates (Figure S1). The presence of degraded DNA in the mucus layer covering the tips of villi was confirmed by imaging thin sections of Carnoy’s solution fixed mucosal tissue stained with DAPI (Figure 2). This type of fixative enables preservation of fragile surface mucus [18], although separation of the mucus layer from the epithelium was observed occasionally, and was most likely caused during processing by different shrinkage rates between the mucus with higher water content than the underlying tissue. The integrity of the epithelial layer of the villi was well preserved. In contrast to the surface mucus, the mucus between villi and close to the crypts (Figure 1C, H) did not appear to contain extracellular DNA.

**Figure 3:**
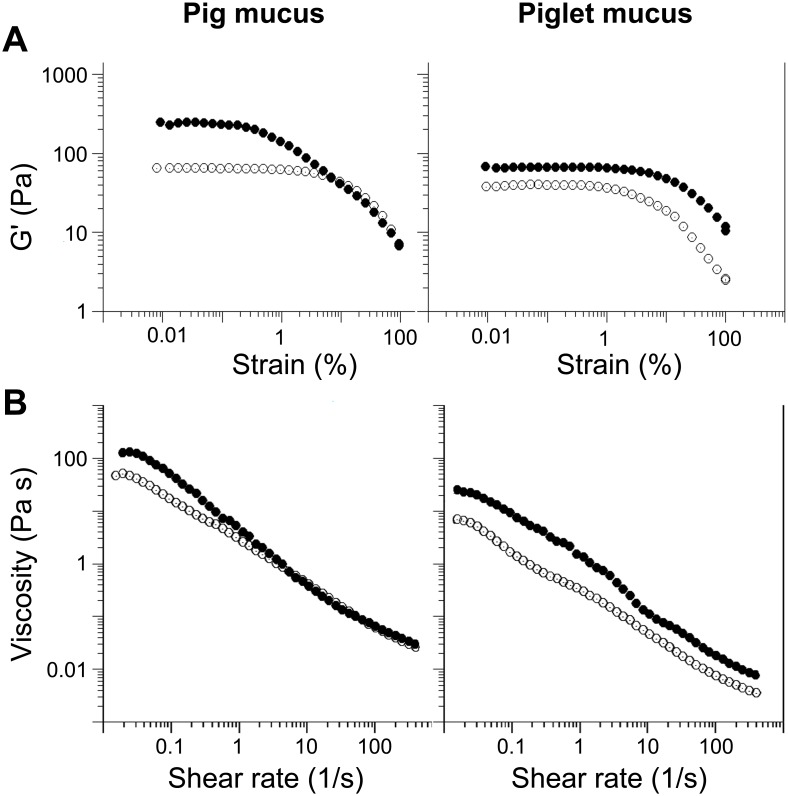
Bulk rheology of the *ex vivo* mucus from pig and piglet. (A) A strain sweep test and (B) a viscosity ramp test for the native mucus (filled circles) and the mucus treated with DNase (open circles). All measurements were done at 37±0.1°C.

**Figure 4:**
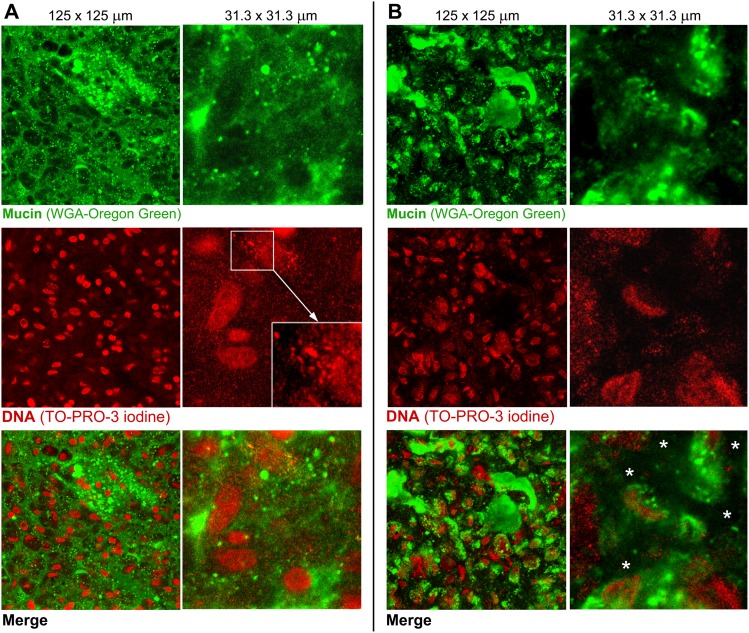
Comparison of the *ex vivo* mucus structures from pig and piglet. Confocal microscopy images of the *ex vivo* small intestinal (jejunal) mucus from (A) pig and (B) piglet, acquired at two magnifications. The upper images show a green channel for mucin stained with WGA-Oregon green, the red-channel images show DNA stained with TO-PRO-3 iodine, and the bottom images are merged views of the two channels. The magnified image (A, DNA staining) highlights a progressive degradation of nuclear DNA into fine particulates. The white asterisks (B, merge) indicate the areas in the piglet mucus with apparent lower local amounts of mucin and DNA as compared to the adjacent aggregates of the two polymers.

In additional studies, we only used the mucus that was directly exposed to the lumen of the intestine (i.e. representative of that shown in Figures 1D, E, I, J, 2 and S1) as it was assumed to represent the first protective layer in the intestine any particulates/nutrients from the lumen have to cross in order to be transported to the epithelium. The mucus was gently removed from freshly excised tissue as explained in the experimental section. Mucus samples were collected from the proximal jejunal regions of the small intestine that was in the absence of digesta. The scraped mucus is referred to as *ex vivo* mucus throughout the paper. The pH of the *ex vivo* mucus was 6.52±0.31 (n = 4) and 6.59±0.38 (n = 3) for samples collected from pigs and piglets, respectively. Oscillatory rheological measurements revealed that both the pig and the piglet mucus samples show characteristics of a viscoelastic medium (Figure 3A), i.e. a distinctive linear viscoelastic region (LVR) at low strain followed by a steep decrease in the elastic modulus (G’) values upon increasing the strain to a critical value sufficient to break the structure of the mucus and make it flow [19]. The pig mucus showed 4 times higher G’ in the LVR than the piglet mucus. The piglet mucus was also less viscous as confirmed by the viscosity measurements (Figure 3B). Samples from both sources showed shear-thinning characteristics. Measurements of the dry weight content yielded 16.7±0.9 mg/g for the piglet mucus and 18.6±0.8 mg/g for the pig mucus. The 10% lower concentration in the piglet mucus is unlikely to account for the 4-fold reduction in the G’ of the LVR as compared to the pig mucus. This suggests differences not just in the concentration but in the microstructure of the mucus samples. The two types of *ex vivo* mucus were visualized with confocal microscopy (Figure 4). As with the images obtained for the mucus structures in the tissue (Figures 1, 2 and S1), the micrographs of the *ex vivo* mucus samples also showed heterogeneity in the distribution of mucin and DNA. In both pig and piglet mucus samples, the DNA was found at different stages of degradation, ranging from the size of ‘intact’ epithelial nuclei down to suspension of fine submicron-size particulates. Both the large and small DNA particulates appeared to form a network or they held in place by the mucin network as it was not possible to separate them from each other by centrifugation, and both migrated together under the force applied (data not shown). The possibility that the majority of the fine DNA particles originated from epithelial nuclear DNA that was undergoing progressive degradation in the mucus seems to be supported by an exemplar micrograph shown in Figure 4A (red channel), where DNA particles with decreasing sizes seemed to be released from a nucleus and surrounded it in the form of a suspension, although contribution of bacterial DNA to the overall DNA content in the mucus cannot be ruled out. The mucins formed a distinct network in the pig mucus, with mucin granules entrapped in it (Figure 4A, green channel). The network often occupied the same areas to those filled with fine DNA particulates. Together they formed a structure that was very different from the way the piglet mucus was organized. In the pig mucus, both mucin and DNA produced a coherent network, whereas in the piglet mucus the network appeared more heterogeneous and fragmented (Figure 4B), consisting of aggregates with high intensity of the fluorescence, from stained mucins and DNA, surrounded by regions with little fluorescence, meaning that the latter represented areas with lower local concentrations of mucin and DNA. The total concentration of DNA in the piglet mucus was found to be lower as compared to the concentration in the pig mucus (4.8±1.3 mg/g vs. 8.0±0.4 mg/g, respectively). Despite this, there was no significant difference in the net ζ-potential of the piglet mucus compared to adult pig mucus (i.e., −11.4±0.9 mV and −11.1±0.4 mV, respectively; Table 1). For the purpose of the measuring procedure, the mucus samples were gently dispersed in buffer, and the ζ-potential values obtained have been assumed to reflect the net charge that might be expected for the surface of mucus layer.

**Table 1:** The ζ-potential of colloidal particles. Data are presented as means ± SD.

Colloidal particle	DNase treatment	ζ-potential (mV)
Dispersed pig mucus	–	–11.1±0.4
Dispersed pig mucus	+	–11.5±1.1
Dispersed piglet mucus	–	–11.4±0.9
Dispersed piglet mucus	+	–12.0±1.4
500-nm latex beads	n/a	–20.1±1.7
1-µm latex beads	n/a	–18.5±1.1

### Diffusion of Particles in Small Intestinal Mucus: Pig vs. Piglet

**Figure 5:**
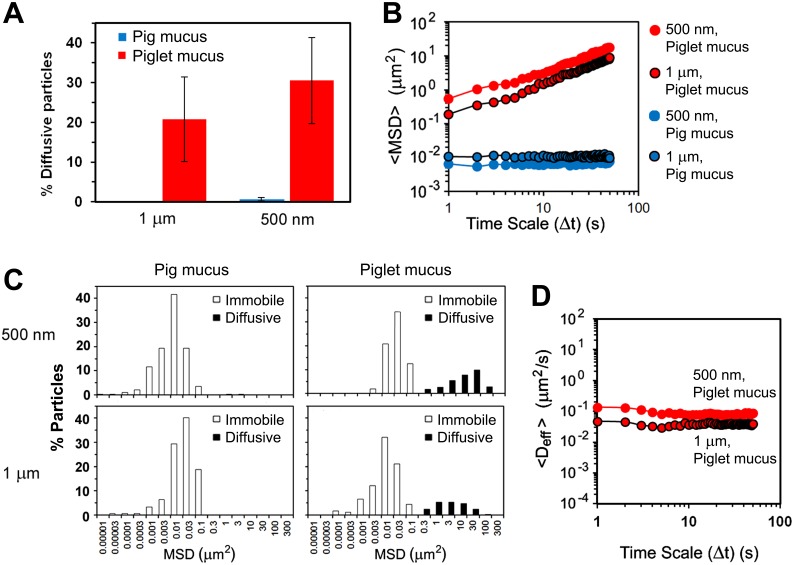
Impact of the mucus structure on diffusion of particles. Transport rates and distributions of 500-nm and 1-µm latex beads in the *ex vivo* small intestinal mucus from pig and piglet: (A) proportions of diffusive particles in the two types of mucus (data shown as mean ± SD), (B) ensemble mean-square displacements (<MSD>) as a function of time scale (Δt) (for the piglet mucus, the <MSD> values calculated for the populations of diffusive particles were only shown), (C) distributions of MSD values obtained for individual beads at the time scale Δt = 50 s, and (D) ensemble diffusivities (<D_eff_>) as a function of Δt calculated for the populations of diffusive particles (n = 3 with 100–150 beads per experiment). All measurements were done at 37±0.1°C.

**Figure 6:**
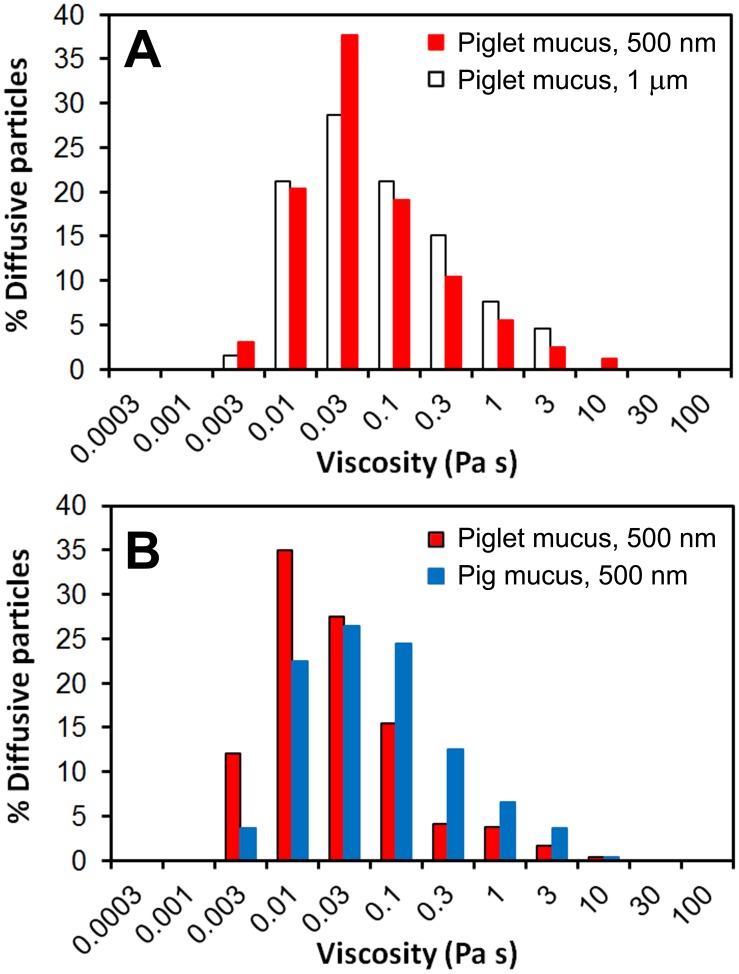
Microviscosity of the mucus. Distribution of the apparent viscosity for the *ex vivo* small intestinal mucus as determined from the motion of 1-µm and/or 500-nm latex beads freely diffusing in the mucus (diffusive fractions; see Figures 5 and 7): (A) results obtained for the native piglet mucus; (B) results obtained for the piglet and pig mucus samples pre-treated with DNase.

The differences in microstructure of the mucus samples from pig and piglet have been further confirmed by particle tracking measurements. The motion of probe particles was monitored with time-lapse confocal microscopy and converted to mean-square displacements (MSD). Two different sizes of carboxylated latex beads, 500 nm and 1 µm (ζ-potential −20.1±1.7 mV and −18.5±1.1 mV, respectively, Table 1), were used to probe the microrheology of the mucus samples. In the pig mucus however, motion of the 1-µm beads was completely obstructed by the mucus network. Only a negligible 0.6% of the total number of the 500-nm beads analyzed was found diffusing over the time scale of the measurements, with the remaining 99.4% being immobile (Figure 5A). This behavior is evident from the ensemble MSD (<MSD>) data, which shows constant values with time (Figure 5B). In contrast, the proportions of diffusive particles in the piglet mucus were ca. 20% and 30% of the total number of 1-µm and 500-nm beads, respectively (Figure 5A). The distributions of MSD values obtained for individual beads after 50 s have been shown in Figure 5C and demonstrate high degree of heterogeneity of the piglet mucus, with distinct fractions of immobile and diffusive particles. The MSD data of individual particles have been converted to diffusion coefficients (effective diffusivities, D_eff_) and finally ensemble D_eff_ (<D_eff_>) calculated for populations of particles expressing free diffusion. Those mobile particles showed constant diffusion in time with <D_eff_> values of 0.039±0.003 µm^2 ^s^−1^ for the 1-µm beads and 0.084±0.012 µm^2 ^s^−1^ for the 500-nm beads (Figure 5D). The D_eff_ data obtained for freely diffusing particles were further used to calculate apparent local microviscosity of the *ex vivo* piglet mucus using the Stokes–Einstein equation. The calculations returned the mean viscosity of 13 mPas for the 500-nm beads, and slightly increased value of 15 mPas for the 1-µm beads. However, the distribution of viscosities displayed by diffusing beads was very broad, stretching from a little more than the viscosity of water up to four orders of magnitude higher, 3 mPas–10 Pas (Figure 6A). The distribution patterns were similar for both sizes of latex beads used, and further highlight the significant heterogeneity of the piglet mucus in the regions where diffusion of the particles could be detected.

### Contribution of the Extracellular DNA to the Microrheology of Mucus

**Figure 7:**
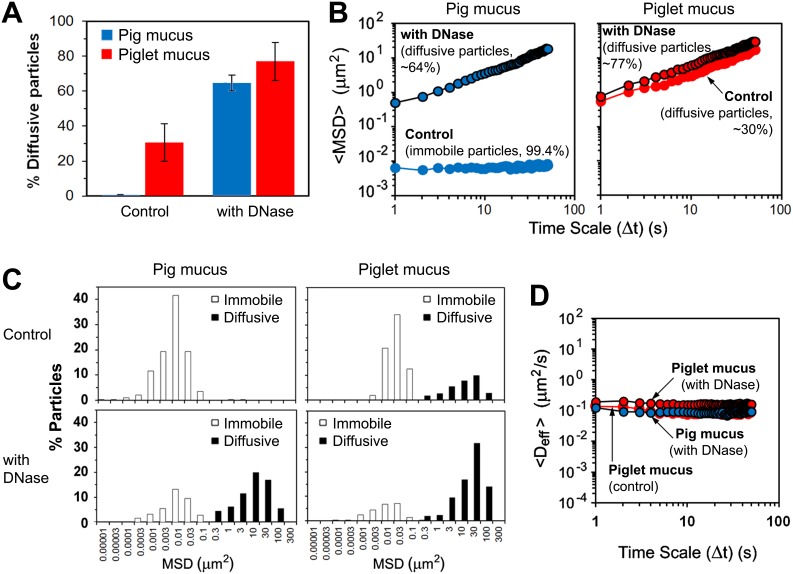
Effect of the extracellular DNA on diffusion in the mucus. Impact of the DNase treatment of the *ex vivo* intestinal mucus from pig and piglet on transport rates and distributions of 500-nm latex beads: (A) proportions of the diffusive particles in the two types of mucus (data shown as mean ± SD), (B) Ensemble mean-square displacements (<MSD>) as a function of time scale (Δt), (C) distributions of MSD values obtained for individual beads at the time scale Δt = 50 s, and (D) ensemble diffusivities (<D_eff_>) as a function of Δt calculated for the populations of diffusive particles (n = 3 with 100–150 beads per experiment). All measurements were done at 37±0.1°C.

As mentioned above, the mucus collected from the small intestine contained DNA at different stages of degradation, with DNA aggregates varying in size. It has been assumed that the network of degraded extracellular DNA, with deformed/elongated structures and fine particulates (Figures 2D, 4 and S1), is able to contribute to the microviscosity of the mucus whereas the DNA still retaining the original shape and size of epithelial cell nuclei can mainly impact on the bulk viscosity by contributing as an excluded volume. In order to evaluate the effect of the extracellular DNA on the diffusion of particles in the mucus, samples of mucus were pre-treated with DNase and results of the transport experiments compared to those obtained for untreated samples. The DNase treatment had no effect on pH of the samples (data not shown). The degradation of DNA resulted in lower G’ and viscosity values recorded for mucus samples in bulk rheology measurements (Figure 3). It also significantly enhanced permeability of mucus to the probe particles (Videos S1 and S2). In the pig mucus, the fraction of diffusive 500-nm latex beads increased from 0.6% to 64%, whereas in the piglet mucus the number of diffusing beads increased to 77% from the initial value of ca. 30% recorded for the untreated mucus (Figure 7A). The <MSD> values increased by nearly two orders of magnitude over 50 s (Figure 7B), although the distance travelled by individual particle still differed significantly (Figure 7C). Again, the MSD data were converted to D_eff_, and finally <D_eff_> calculated for populations of particles expressing free diffusion (Figure 7D). The latex beads diffused in the DNase-treated pig mucus with a constant value of 0.088±0.009 µm^2 ^s^−1^. Normal diffusion was also observed in the piglet mucus. However, apart from the significant increase in the number of diffusive particles, the mean diffusion was twice as fast as in the mucus before the DNase treatment, with the <D_eff_> values increasing from 0.084±0.012 µm^2 ^s^−1^ to 0.163±0.010 µm^2 ^s^−1^ (Figure 7D). The diffusion data were used to calculate local microviscosity experienced by individual freely diffusing particles. In both pig and piglet mucus samples treated with DNase, the distribution of viscosity values was very broad, ranging from 3 mPas to 10 Pas (Figure 6B). In the piglet mucus however, there was a shift towards lower viscosities as compared to the mucus not treated with DNase (Figure 6A), with the mean viscosity values of 13 mPas and 7 mPas, before and after the treatment, respectively. The mean viscosity calculated for the DNase-treated pig mucus was 13 mPas.

## Discussion

We have shown that intestinal mucus contains significant amounts of DNA that largely derives from shed epithelial cells and contributes to the mucus viscoelastic properties. The small intestine of humans, like other mammals, is covered by a single layer of epithelial cells that is renewed typically every 3–5 days [10]. For example, in the mouse about 1400 cells are shed from each villus per 24 hours [20]. Such fast turnover rates suggest that about 10 ng of DNA from the shed cells of each villus can be accumulated in the mucus layer lining the intestine and therefore contribute to the rheological properties and permeability of the layer, although this is the first time that it has been shown.

DNA has previously been reported to increase the viscosity of other types of mucus secreted in the body. For example, in cystic fibrosis, large amounts of DNA released from numerous neutrophils involved in the response to inflammation have long been known to contribute to the enhanced viscosity of the respiratory mucus [21], [22]. The resulting pathological decrease in the mucociliary transport of accumulated purulent secretion can be overcome by applying DNase. The addition of bovine pancreatic DNase [23] and, more important in clinical practice, highly purified recombinant human DNase I [24], [25] were found to significantly reduce the viscosity of the cystic fibrosis mucus, and thus improve its transportability.

In the above example, increased amount of DNA seen in secreted mucus was a result of the immune-response to the pathology, whereas in the small intestinal mucus studied here, the presence of DNA is a consequence of intestinal epithelium turnover. We observed accumulation of nuclear DNA in the mucus adjacent to the tips of villi but not in the mucus located between villi towards the crypts. This is because the majority of the cells are shed from the villus tips [11], [12], where they are trapped by the overlying mucus layer. Apoptosis subsequent to shedding [14] in an environment rich in digestive enzymes may lead to the cellular debris being quickly degraded leaving behind the nuclei, which are apparently more slowly digested. This could account for the observed accumulation of fragmented DNA in the uppermost regions of the mucus layer that is directly exposed to the intestinal lumen, and therefore must serve as a primary physical barrier to pathogens while allowing nutrient transport to the underlying epithelium. The lower values of the elastic modulus and the viscosity found for the piglet mucus were probably caused by differences in local concentrations of DNA and mucin compared to the pig mucus. Recently, Georgiades et al. [26] showed that purified porcine small intestinal MUC2 mucin can undergo a sol-gel transition if the pH is reduced from 7 to 1. However, the differences in rheological properties of the *ex vivo* mucus samples used in our studies cannot be explained by variations in pH since for both pig and piglet samples similar pH values (pH 6.5–6.6) were recorded. Those authors also showed [26] that viscosity of the intestinal mucin at neutral pH, characteristic of the small intestinal environment, was two orders of magnitude smaller than its viscosity at pH 1 or the viscosity of the stomach MUC5AC mucin gelled at low gastric pH. This supports our finding of a significant role of the extracellular DNA in determining viscosity and viscoelasticity of the small intestinal mucus under physiological conditions. Despite fairly similar dry mass content between the two types of mucus, the mucin and DNA in the pig mucus formed a network, which appeared more coherent at the micro-scale than that seen in piglets. Only in the piglet mucus did the 500-nm and 1-µm latex beads show significant levels of mobility. The piglet mucus appeared to be comprised of aggregates of mucin and DNA surrounded by regions with lower local concentrations of the two polymers, and this is where the majority of diffusing probe particles were observed. The increased permeability of the piglet mucus might have been caused by the lower total DNA content, which in turn may have originated from a slower turnover of the intestinal epithelial cells in neonates. As reported by others [17], the cellular organization of the early neonatal crypts, generating epithelial cells in the small intestine, is different from that seen in adults. This could result in the neonatal villus enterocytes having a longer life span than adult enterocytes. In new-born mice, the epithelial cell turnover has been found to be between 7 and 11 days [27], [28] as compared to maximum 3 days in adult mice [29]. Schmidt et al. [17] reported that development of the intestinal epithelium in mice, leading to mucosal architecture characteristic of the adult epithelium, took about 14 days after birth. During this time, the villi elongate, reaching a length equivalent to that of adult villi by 20–24 days after birth [29]. In the new-born piglets, the rate of renewal of the small intestinal villus epithelium is also decreased. The turnover time was reported to range from 7 to 10 days [16], [30] but after 3 weeks it can be reduced to no more than 4 days as the intestinal crypts and villi mature [16]. Apart from the stage of development of the intestinal crypts, the cellular turnover rate and, as a consequence, the mucus composition can be influenced by the ratio of the number of crypts to the number of villi in the small intestine. In general, the ratio increases over the first several weeks after birth. In 3-week old mice the ratio of about 4.5 was reported [28], which is considerably lower than the adult value of about 11 crypts per villus [31]. Similar increase in the ratio with age was shown in rats [32]. This may account for the shorter turnover time of epithelial cells in adult mammalian small intestine, and hence increased amounts of DNA in the adult intestinal mucus. This supports the lower concentration of DNA in the small intestinal mucus of 2-week old piglets as compared to mature pigs observed in this study.

In addition to differences in rates of cellular turnover, the distribution of mucin-producing goblet cells and the glycation of the mucin produced vary in the gastrointestinal tract during postnatal development in all mammalian species, including humans [33]. The ratio of neutral to acidic mucins generally increases between birth and the weaning period and decreases after weaning. The colonic mucins of new-born pigs were found highly sulphated and sialylated [34]. The presence of acidic mucins in early life was suggested to be of particular importance as an innate defense barrier because the acquired immune system is not fully functional in the neonatal intestine [33]. Factors such as diet [34], [35] and intestinal microbiota colonization [33], [36] have been shown to significantly affect the development of intestinal mucin production after birth. Although the literature addressing the developmental pattern of mucin production in the neonatal intestine is limited and mainly focused on colonic mucins [34]–[38], similar rules may apply to the small intestine, especially as the MUC2 mucin is the major secreted mucin in both the colon and the small intestine [39].

The progressive change in cellular turnover of the intestinal epithelium and in mucin production correlates with our finding of the contrasting structural organization of DNA and mucin in the intestinal *ex vivo* mucus obtained from piglet and pig that have been used in this study as models of infant and adult human intestinal mucus. The resulting differences observed in macro- and microrheological properties of the mucus between the two age groups may also lead to an age-specific permeability of the mucus to luminal contents such as partially digested food particulates or bacteria. Apart from the variations in mucus structure, other factors such as higher concentrations of digestive enzymes and bile salts in the adult human small intestinal environment [40] can play an important role in colloidal transport through the mucus layer. We recently showed that adsorption of bile salts to particles, including partially digested lipid droplets, was crucial in their transport through the mucus [7], [8]. However, more work is required to ascertain whether differences in concentration of these physiological surfactants between adult and infant intestinal conditions may affect transport characteristics in the mucus.

To our knowledge, this is the first time that a comparison of the structural organization of the protective intestinal mucus between different age groups has been reported and important physiological differences pointed out. To date, scientific literature has focused on the role of mucin glycoproteins as major gel-forming components of mucus in the gut [1], [2], [9], [39]. In this work, we present evidence for a significant contribution of the extracellular DNA to barrier function of the small intestinal mucus. The extracellular DNA from shed epithelial cells significantly contributed to the viscosity of the small intestinal mucus, which in turn affected its permeability as revealed by measurements on the DNase-treated samples. However, the effect seemed to be greater in the adult pig mucus than in the piglet mucus due to the lower amounts of DNA and more heterogeneous structure of the latter. Our results highlight the importance of extracellular DNA in increasing the physical barrier properties of the mucus, at least in the small intestine where the cellular turnover is rapid and where the mucus layer is thinner than in the colon or the stomach [5], [41]. This work also provides insight into the changes in small intestinal mucus barrier function as the gut matures. Such knowledge has implications for the oral delivery of nutrients, bioactives and pharmaceuticals for different age groups. Our results can also have a potential biomedical importance as alterations in mucus composition and structure appear to characterize many diseases of gastrointestinal mucosal tissues.

## Materials and Methods

### Ethics Statement

Piglet small intestines were obtained from INRA-UMR PEGASE (Physiology, Environment and Genetics for the Animal and Livestock Systems) in Saint-Gilles, France, in strict accordance with the recommendations of the French Ministry of Agriculture (Directive 2001-464 29/05/01) and EEC (Directive 86/609/EEC) for the care and use of animals in research, and under approved authorization certificate for experiments on animals (certificate no. 7676). INRA-UMR PEGASE is a holder of the agreement for experimentation on pigs (no. A35622) authorized by the Veterinary Services of the French Ministry of Agriculture. Piglets were reared and slaughtered in compliance with French national regulations and according to procedures approved by the French Veterinary Services at INRA-UMR PEGASE. Piglets were slaughtered by electrical stunning and exsanguination at the experimental slaughterhouse that is authorized for commercial meat production. As the piglets used in this study were not specifically included in any experimental protocol on living animals before slaughtering, there was no ethical requirement for collecting intestinal tissue samples from slathered animals. Pig small intestines were obtained from a local authorized slaughterhouse in Norwich, UK (H. G. Blakes Ltd, Cotessey, Norfolk, UK), from slaughtered healthy animals, as a part of a commercial meat production process, and therefore any ethical requirements that would be specific for this part of the study were not required. The authors obtained permission from the slaughterhouse to use these animal parts in the study.

### Materials

Red fluorescent carboxylate-modified latex beads (Sigma, Poole, UK; L3280 500-nm and L5405 1-µm diameters; 2.5% w/v aqueous dispersions) were diluted to 0.125% w/v or 0.25% w/v, respectively, with PBS buffer (Sigma, Poole, UK; pH 7.4). All suspensions were incubated at RT for 20 min prior to use. DNase I from bovine pancreas (2000 Kunitz units per mg protein; Sigma, Poole, UK; D4513) was dissolved in PBS buffer containing 5 mM MgCl_2_, pH 7.4, to give the enzyme concentration of 20 mg/mL and activity 40 k Kunitz units per mL. Small aliquots of the enzyme stock (20 µL) were frozen in liquid nitrogen and stored at −80°C prior to use.

### Small Intestinal Mucus and Tissue Samples

Two sources of mucus from freshly excised porcine proximal small intestine (jejunum) were used in this study: the mucus obtained from 7–10-month old, mature pigs (it has been referred to as the *ex vivo* pig mucus throughout the paper) and the mucus from 2-week old piglets fed exclusively on mother’s milk (this has been referred to as the *ex vivo* piglet mucus). For both pigs and piglets, immediately after slaughter, the segment of the GI tract containing the whole small and large intestines was removed. Only the proximal jejunum (from the first meter of the small intestine after the duodenum, and free of contents) was used in further procedures. Several short segments of the jejunum (ca. 3 cm long) were either (i) immediately frozen in liquid nitrogen and stored at −80°C prior to use, or (ii) immediately placed into Carnoy’s fixative solution [18] for 2 hours followed by storage in 70% ethanol until processing and paraffin embedding. Alternatively, the fresh jejunal segments were carefully opened longitudinally and the mucus gently removed from the mucosal surface with a plastic scraper (Corning, NY), and within 30 min from slaughter. Special attention was paid during the process to avoid damaging the tissue. Small aliquots of the scraped mucus (*ex vivo* mucus) were immediately transferred to 0.5-mL plastic tubes, frozen in liquid nitrogen and stored at −80°C. Aliquots were incubated for 10 min at RT prior to use in experiments. As found previously [7], the freezing procedure did not significantly affect rheological properties of the mucus.

### DNA Concentration in Mucus

In order to determine the concentration of DNA, *ex vivo* mucus samples were diluted 10 fold in buffer (0.15 M NaCl, 15 mM tri-sodium citrate) then 0.2-mL aliquots were mixed with 0.4 mL of a solution of diphenylamine (5 g in 500 mL of glacial acetic acid, 13.75 mL of concentrated sulphuric acid). The samples were heated in a boiling water bath for 10 min, allowed to cool and their absorbance measured in triplicate on 0.2 ml against a blank at 600 nm in a micro titre plate using a plate-reader. A calibration curve consisting of salmon sperm DNA (Sigma, Poole, UK) was used to determine the actual sample DNA concentration.

### Dry Weight Determination

Aliquots of the *ex vivo* mucus (ca. 100 mg) were placed in pre-weighed, dry aluminum pans. The weight of the pan with the wet sample was recorded to 1 µg using a Mettler ME30 balance. The samples were dried in an oven heated to 65°C for at least 24 h followed by cooling in a desiccator for at least 30 min prior to weighing. Samples were placed back into the oven, and the cycle was repeated until successive weight differences were less than 3%. All samples were analyzed in triplicate, and means of the results used for further analysis.

### Confocal Microscopy of Fixed Intestinal Tissue

Thin (4-µm-thick) cross-sections of Carnoy’s fixed jejunal segments were stained with DAPI for DNA, and with Alexafluor-633 conjugated wheat germ agglutinin (WGA-Alexa633) to visualize mucins. Slides were mounted using Fluoroshield media (Sigma, Poole, UK) to preserve fluorescence. Mucus structure was visualized using Leica SP5 laser scanning microscope (Leica Microsystems, Mannheim, Germany).

### Confocal Microscopy of Unfixed Intestinal Tissue and *ex vivo* Mucus

Images were taken with a Leica TCS SP confocal laser scanning head mounted on a Leica DMRE microscope (Leica Microsystems (UK) Ltd, UK). Fluorescence from the sample was excited at 488 nm and 633 nm. The unfixed, frozen segments of the small intestine were sectioned to obtain thick (ca. 2 mm) cross-sections of the intestine. The sections were carefully placed in a laboratory-made 2-mm thick optical chamber mounted on a standard microscope slide and stained at RT for 5 min for mucins using wheat germ agglutinin-Oregon Green 488 conjugate (WGA-Oregon Green; Invitrogen W6748, 1 mg/mL in 0.1 M sodium bicarbonate buffer, pH 8.3) and for DNA with TO-PRO-3 Iodine (1 mM solution in DMSO; Invitrogen, Paisley, UK) at the final concentrations of the dyes of 10 µg/mL and 5 µM, respectively in PBS buffer (pH 7.4) used to fill the chamber. Subsequently, the staining buffer was gently removed and replaced with fresh PBS buffer, and the chamber covered with a cover slip. Images were acquired at RT within a maximum of 25 min, 40–60 µm below the surface of specimen. Eight scans were averaged during the creation of each image. Imaging of the *ex vivo* mucus samples was done in a similar way after staining the mucus for mucins and DNA with small quantities of the stock solutions to give the final concentrations of the stains in the mucus as above and to avoid diluting the mucus.

### Multiple-particle Tracking in *ex vivo* Mucus

The *ex vivo* mucus samples were stained for 5 min at RT with WGA-Oregon Green to the final dye concentration of 10 µg per 1 mL sample. The mucus was then mixed gently with a small amount of the diluted suspension of fluorescent latex beads (mucus:suspension, 99∶1, v/v) and placed in a 300-µm thick optical cell. In the experiments on DNase-treated mucus, the suspension of beads contained the DNase to give the final enzyme concentration in the mucus of 400 Kunitz units per mL. Specimens were carefully covered with a coverslip, placed on a temperature-controlled microscope stage (37±0.1°C) and incubated for 20 min prior to particle tracking experiments. Each specimen was used for up to 30 min. Motion of the beads in the mucus was recorded at 37±0.1°C with the confocal microscope equipped with a ×40 oil-immersion objective, NA 1.25, and at a time resolution of 1 s over 50 s. The pinhole size was increased to 2 Airy units in order to facilitate the extended tracking of particles in the focal plane. Specimens were scanned 40–60 µm below the coverslip. The number of tracked beads was typically kept in the range of 5–20 per field of view in order to avoid their mutual interactions. Trajectories of 100–150 beads per experiment were analyzed. Experiments were performed in triplicate for each condition. Measurements were carried out on the intestinal *ex vivo* mucus obtained from three individual animals for each group. The results are shown as means ± SD and/or distribution of data from individual measurements. The trajectories were analyzed by using Image-Pro Analyzer 7.0 software (Media Cybernetics Inc., Silver Spring, MD) and are 2D representations of a 3D transport. Movement of an individual particle centroid was transformed into time-dependent mean-square displacement (MSD), <Δr^2^(Δt)> = <Δx^2^+ Δy^2^>, where Δx and Δy are particle displacements in x and y directions, respectively, and Δt is the time-scale over which the displacement was calculated [42]–[44]. By averaging MSDs with identical Δt from trajectories of many particles, ensemble mean-square displacement <MSD> for families of particles was calculated. Effective diffusivities (diffusion coefficients, D_eff_) were obtained from D_eff_ = MSD/4Δt, and ensemble effective diffusivities <D_eff_> calculated for families of particles by averaging D_eff_ values obtained for individual particles. For the particles that were undergoing simple diffusion, apparent local microviscosity of *ex vivo* mucus samples (η) was calculated using the Stokes–Einstein equation, D = k_B_T/6πηr, were D is the diffusion coefficient independent of time, k_B_ is the Boltzmann’s constant, T is an absolute temperature in Kelvin and r is the radius of diffusing particle.

### Bulk Rheology

Rheological properties of *ex vivo* mucus samples were investigated in dynamic oscillatory and rotational tests using a controlled strain AR2000 rheometer (TA Instruments, Crawley, West Sussex, UK) equipped with a cone and plate geometry (aluminum cone; 6°/20 mm, the cone angle/diameter). A thin layer of low-viscosity silicone oil (Dow Corning 200/20 cs) was gently spread over the sample edge in order to prevent water evaporation. The following tests were performed at 37±0.1°C: (i) a strain sweep test at fixed frequency (1 Hz) where the strain amplitude was increased stepwise from 0.01 to 100% over a period of ca. 4 min, (ii) a viscosity ramp test for a shear rate being increased from 0.01 to 500 s^−1^ over 15 min. Measurements were done for control (native mucus) and DNase-treated samples (mucus:DNase solution, 99∶1, v/v, to the final enzyme concentration in the mucus of 400 Kunitz units per mL, gently mixed and incubated at 37°C for 20 min prior to measurement). In the control samples, the DNase solution was replaced with PBS buffer.

### ζ-potential Measurements

The ζ-potential of dispersions of particles (i.e. dispersed mucus, latex beads) was obtained from dynamic light scattering measurements at 37°C using a Nano-ZS Zetasizer (Malvern Instruments Ltd, Malvern, UK). Prior to analysis, *ex vivo* mucus samples (both, the non-treated controls and the DNase-treated) were gently dispersed in PBS buffer (pH 7.4) to give a concentration of ca. 0.1% w/v. The latex dispersions were diluted in PBS buffer to the concentration of ca. 0.001% w/v. Diluted dispersions were then injected into a DTS1060 folded capillary cell (Malvern Instruments Ltd). Each sample was analyzed at least 20 times and the results displayed as a mean. Data shown are the mean and standard deviation from three dispersions prepared under the same conditions.

## Supporting Information

Figure S1 **Fragmented DNA in pig small intestinal (jejunal) mucus.** Confocal microscopy showing TO-PRO-3 iodine staining for DNA in unfixed intestinal mucus aggregates located above the tips of the villous mucosa and exposed to the intestinal lumen. The image is an average of eight scans (scale: 31.3 µm × 31.3 µm).
(TIF)

Video S1 **500-nm latex beads immobilized in native pig small intestinal (jejunal) mucus (200 s shown at 10× speed).**
(WMV)

Video S2 **Transport of 500-nm latex beads in pig small intestinal (jejunal) mucus over the course of 200 s (shown at 10× speed).** The mucus has been pre-treated with DNase. The Brownian movement of most beads is unhindered.
(WMV)
