# Supplementary figures and images for: Transport of Particles in Intestinal Mucus under Simulated Infant and Adult Physiological Conditions: Impact of Mucus Structure and Extracellular DNA
Source: PLoS One. 2014 Apr 22;9(4):e95274. doi: 10.1371/journal.pone.0095274 (PMC3995753; doi:10.1371/journal.pone.0095274)

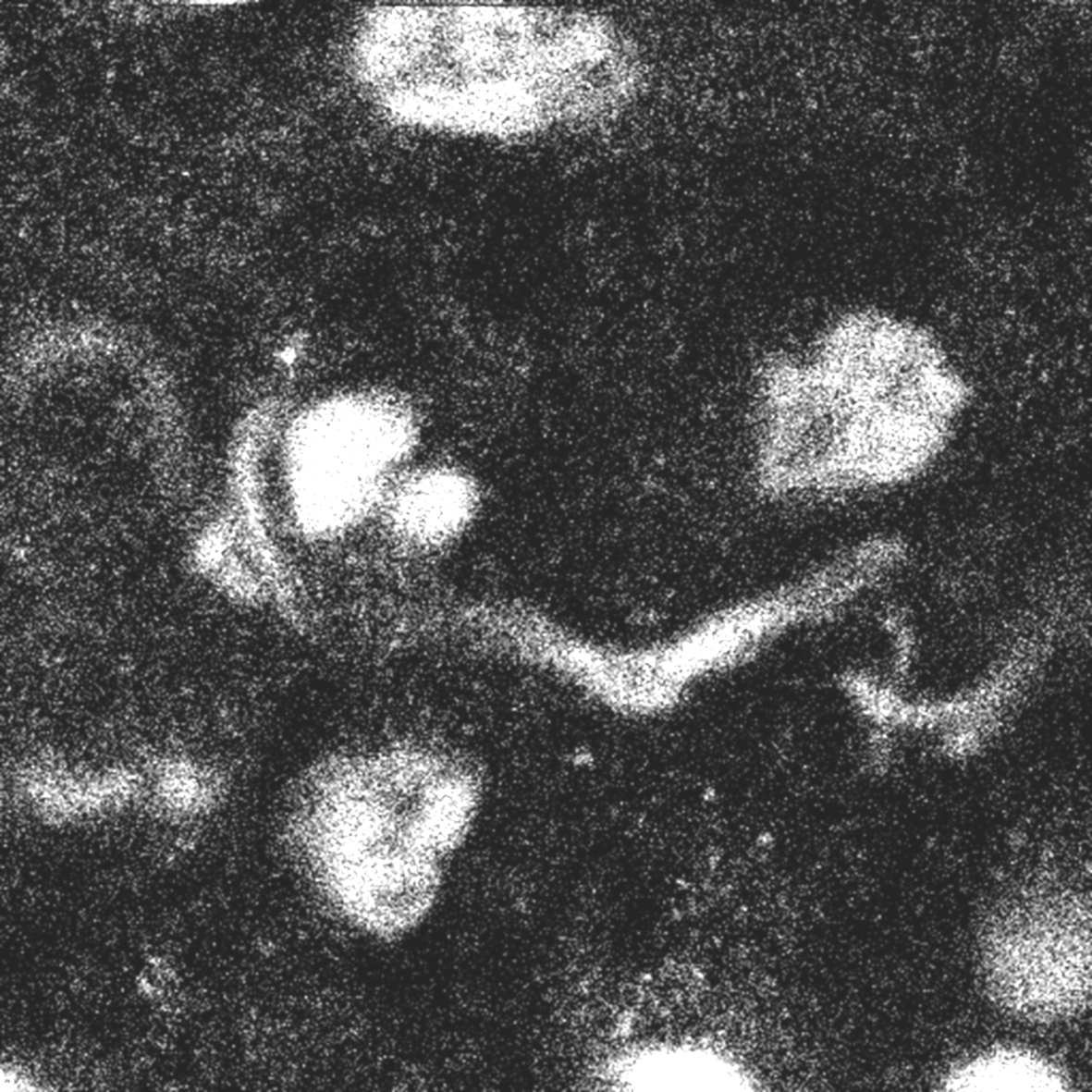

Supplement: Figure S1 — Fragmented DNA in pig small intestinal (jejunal) mucus. Confocal microscopy showing TO-PRO-3 iodine staining for DNA in unfixed intestinal mucus aggregates located above the tips of the villous mucosa and exposed to the intestinal lumen. The image is an average of eight scans (scale: 31.3 µm × 31.3 µm). (TIF) [file pone.0095274.s001.tif]
